# Draft Genome Sequence of *Flavobacterium* sp. 316, a Baltic Sea Isolate Exhibiting a High Level of Resistance to Marine Stress Conditions

**DOI:** 10.1128/genomeA.00180-16

**Published:** 2016-03-31

**Authors:** Joanna Karczewska-Golec, Maja Kochanowska-Łyżen, Paweł Olszewski, Magdalena Bałut, Marta Moskot, Arkadiusz Piotrowski, Piotr Golec, Agnieszka Szalewska-Pałasz

**Affiliations:** a Department of Molecular Biology, University of Gdańsk, Gdańsk, Poland; b Department of Biology and Pharmaceutical Botany, Medical University of Gdańsk, Gdańsk, Poland; c Laboratory of Molecular Biology (affiliated with the University of Gdańsk), Institute of Biochemistry and Biophysics, Polish Academy of Sciences, Gdańsk, Poland

## Abstract

Here, we present the draft genome sequence of *Flavobacterium* sp. 316, isolated from brackish water of the Gulf of Gdańsk, southern Baltic Sea. The assembly contains 3,971,755 bp in 17 scaffolds. The sequence will facilitate postgenomic studies on bacterial stress responses in the challenging habitat of the Baltic Sea.

## GENOME ANNOUNCEMENT

*Flavobacterium* sp. 316 was isolated in 2005 from the surface water of the Gulf of Gdańsk (54°33′01.06″N, 18°39′45.50″E), a southeastern bay of the Baltic Sea. Phylogenetic analyses based on 16S rRNA gene sequence comparisons revealed a clear affiliation with the family *Flavobacteriaceae* and indicated 97% 16S rRNA gene sequence identity with *Flavobacterium jumunjinense* and *F. maris.*

Members of the genus *Flavobacterium* have been isolated from diverse habitats (soil, mud, freshwater and marine environments, including diseased freshwater and marine fish) that are widely separated geographically (1). This diversity of lifestyles and habitats is also reflected in the case of *Flavobacterium* sp. 316, which was isolated from the Baltic Sea—one of world’s largest brackish water environments with local, dynamic shifts in salinity*,* mainly due to varying freshwater inflows (2, 3). Molecular mechanisms enabling brackish water bacteria to survive in this challenging niche are poorly understood. This inspired us to determine the draft genome sequence of *Flavobacterium* sp. 316 as a groundwork for pursuing more detailed postgenomic analyses of stress response in the isolate.

Whole-genomic DNA was purified with the use of the Sigma GenElute bacterial genomic DNA kit. Two libraries, a shotgun library and a 3-kb library, were constructed and then sequenced using the Illumina HiSeq 2000 platform, generating a total of 31,918,348 reads, which were analyzed and quality-checked using FastQC version 0.10.1 (http://www.bioinformatics.babraham.ac.uk/projects/fastqc). Low-quality data were then filtered such that, for a pair of paired-end reads, each read had more than 90% of bases with a quality score greater than or equal to Q20. After error correction, a total of 25,247,316 high-quality reads were retained, resulting in a 311× average genome coverage. *De novo* assembly was performed on the SOAPdenovo2 platform (4) at optimal *k*-mers (counted with the use of JELLYFISH version 1.1.10) (5).

The final assembly consisted of 17 scaffolds, covering a draft genome size of 3,971,755 bp, with an *N*_50_ scaffold length of 537,004 bp. The minimum and maximum scaffold lengths were 1,015 and 787,447 bp, respectively. The draft genome was annotated using the NCBI Prokaryotic Genome Annotation Pipeline (PGAP) version 3.0 (6). The annotation revealed 3,485 predicted protein-coding sequences, 7 rRNA operons, and 51 tRNA genes. The average G+C content of the *Flavobacterium* sp. 316 genome is 29.50 mol%, which is consistent with other sequenced genomes within this genus (1, 7) and close to the value of 28.8 mol% determined for *F. maris* (7). Four prophages were identified in the draft genome using PHAST (8).

Among the predicted proteins, stress response mediators (osmoregulatory porins, superoxide dismutases, DNA alkylation repair proteins, reductases involved in resistance to heavy metal compounds, and a toxin-antitoxin system) and a number of protein secretion and translocation systems were identified. This confirms the results of our preliminary phenotypic analyses, which indicated that *Flavobacterium* sp. 316 is well equipped to confront various stressors in the challenging niche of the Baltic Sea.

### Nucleotide sequence accession numbers.

This whole-genome shotgun project has been deposited at DDBJ/EMBL/GenBank under the accession number JYGZ00000000. The version described in this paper is the first version, JYGZ01000000.
